# Supplementary material for: In Vivo Production of Monoclonal Antibodies by Gene Transfer via Electroporation Protects against Lethal Influenza and Ebola Infections
Source: Mol Ther Methods Clin Dev. 2017 Sep 20;7:74–82. doi: 10.1016/j.omtm.2017.09.003 (PMC5633264; doi:10.1016/j.omtm.2017.09.003)
Supplement: Document S1. Supplemental Materials and Methods and Figures S1–S7 [file mmc1.pdf]

OMTM, Volume 7

Supplemental Information

In Vivo Production of Monoclonal Antibodies by Gene Transfer via Electroporation Protects against Lethal Influenza and Ebola Infections

Chasity D. Andrews, Yang Luo, Ming Sun, Jian Yu, Arthur J. Goff, Pamela J. Glass, Neal N. Padte, Yaoxing Huang, and David D. Ho

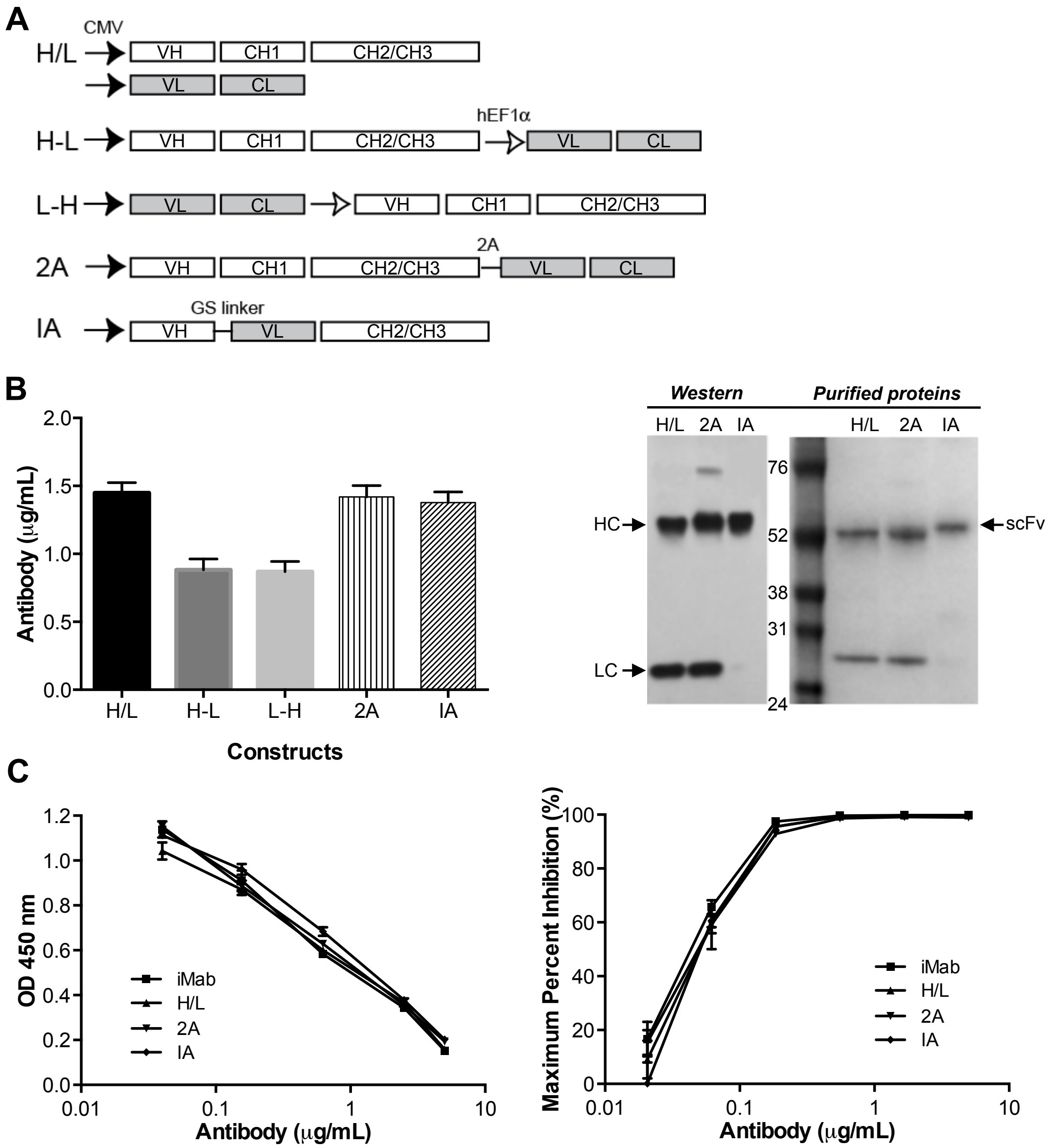

Figure S1

5A8 expression cassettes and *in vitro* characterizations.

(A) Schematic representation of mAb transgene configurations (heavy chain genes shown in white and light chain shown in gray) in the pVAX1 vector. Cytomeglovirus (CMV) and human elongation factor-1 alpha (hEF1α) promoters are indicated by closed black or open white arrows, respectively. The 2A cassette contains H-and L-chain genes separated by a furin cleavage site coupled with a P2A self-processing peptide (2A). The immunoadhesin (IA) contains a single-chain variable fragment (scFv) fused to the Fc region. **(B)** Left panel: *In vitro* 5A8 expression in supernatant 72 hours after pDNA transfection of 293A cells measured by CD4 binding ELISA (mean ± SEM). Right panel: Western blot of cell culture supernatants detected by anti-mouse HRP and mAbs purified from supernatants were resolved by SDS-PAGE and visualized with Coomassie Blue. The faint ~80kDa band detected in the 2A samples by Western blot is unprocessed antibody. Molecular weight ladder represents kDa. **(C)** Left panel: Purified mAbs compete with iMab-HRP for sCD4 binding. Right panel: Purified mAbs neutralize HIV-1 pseudotyped virus tested in TZM-bl cells.

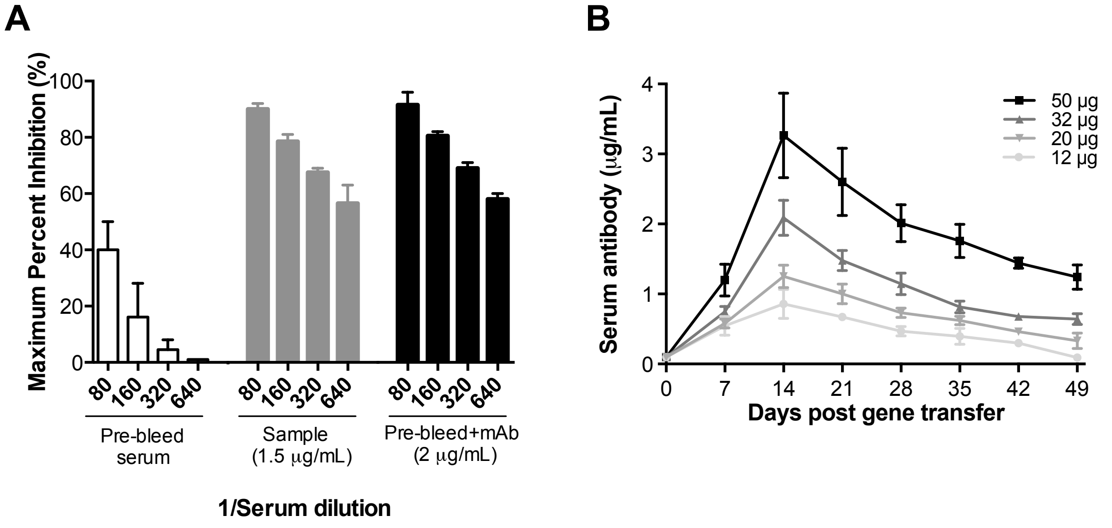

Figure S2

EP delivery of pDNA results in functional dose-dependent serum mAb in mice.

(A) Mice were administered 50 µg H/L 5A8 pDNA in the pVAX1 vector followed by EP. To verify functional activity of the expressed mAb, maximum percent inhibition (MPI) measured by psuedotyped HIV-1 neutralization in TZM-bl cells was compared between serially diluted pre-bleed serum, serum samples taken after DNA/EP, and pre-bleed serum mixed with *in vitro* produced mAb. Mouse serum prior to gene transfer (pre-bleed) was evaluated for antibody neutralization of HIV-1, showing non-specific activity at low serum dilutions. Endogenously expressed mAb retains functional activity comparable to *in vitro* produced mAb. **(B)** mAb expression is pDNA dose-dependent. BALB/c mice (n=6) were injected with 12, 20, 32 or 50 µg of H/L 5A8 pDNA in the pVAX1 vector followed by EP. Serum was collected weekly and mAb concentration was determined by sCD4 binding ELISA. All data are presented as mean ± SEM.

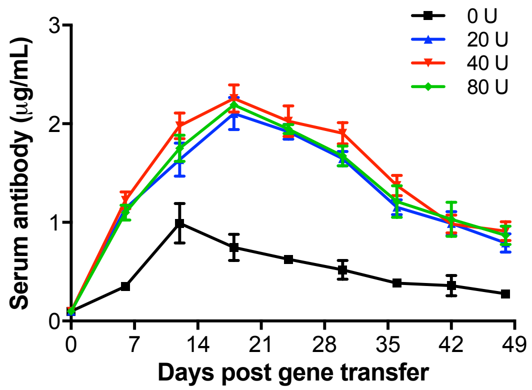

Figure S3

Hyaluronidase pretreatment enhances mAb expression.

BALB/c mice (n=4-5) were pretreated with 20, 40 or 80 U of hyaluronidase or saline (0 U) as a control 2 hours prior to administration of 20 μg of H/L 5A8 pDNA in the pVAX1 vector followed by EP. Serum was collected weekly and mAb concentration was determined by sCD4 binding ELISA. Antibody expression was evaluated by area-under-the curve measurements during the 48-day period studied. All data are presented as mean ± SEM.

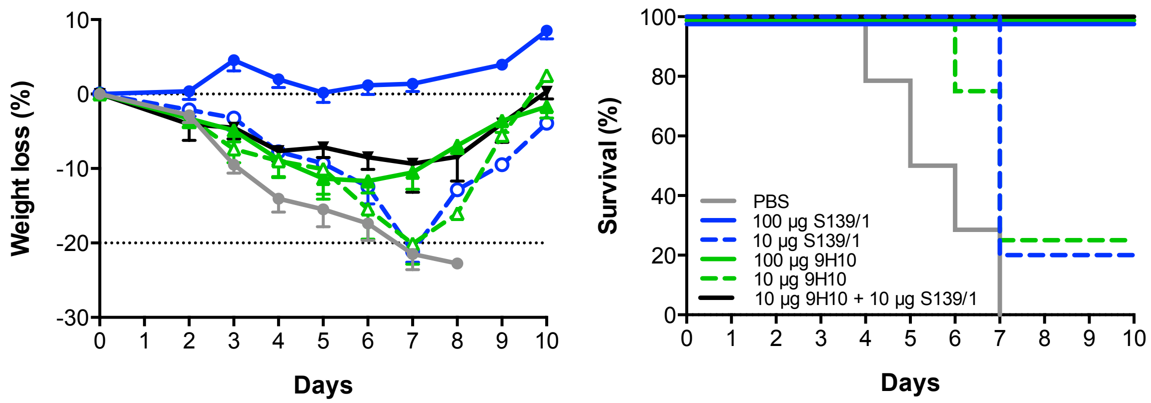

Figure S4

Passive administration of anti-influenza mAbs in mice defines the partially protective dose against influenza challenge.

BALB/c mice (n=3-5) were passively administered mAb via the intraperitoneal route one day prior to influenza challenge. Mice were challenged on day 0 with 13 MLD_{50} of H3N2 (A/Aichi/2/68) delivered intranasally. Left panel: mean weight loss (%) ± SEM compared to day 0. Right panel: survival (%) as depicted in Kaplan-Meier plots following challenge.

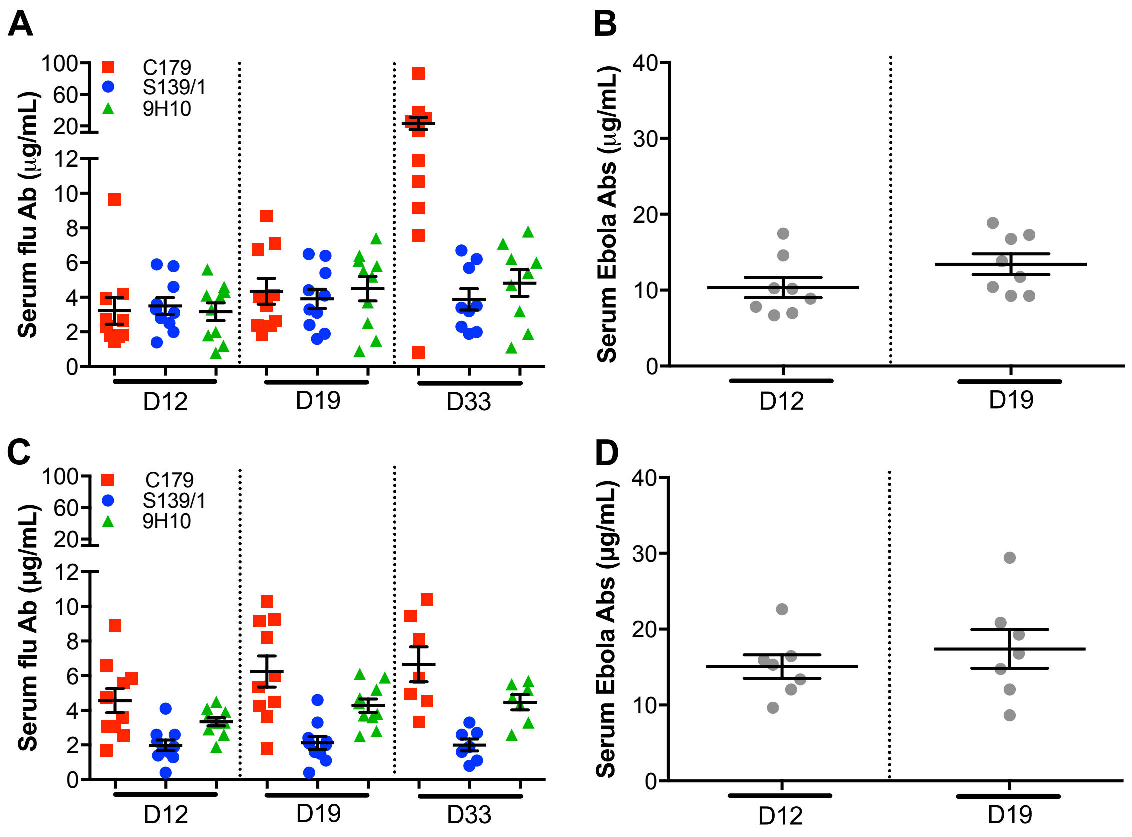

Figure S5

Serum mAb concentrations from mice challenged with group 1 or group 2 influenza viruses.

Mice were administered pDNA followed by EP and challenged as described in Fig. 3. mAb expression from mice challenged with **(A-B)** H1N1 and **(C-D)** H3N2. **(A, C)** Individual mAb concentrations for C179, S139/1 and 9H10. **(B, D)** Total anti-Ebola mAb concentrations. Each symbol represents an individual animal and error bars indicate mean ± SEM.

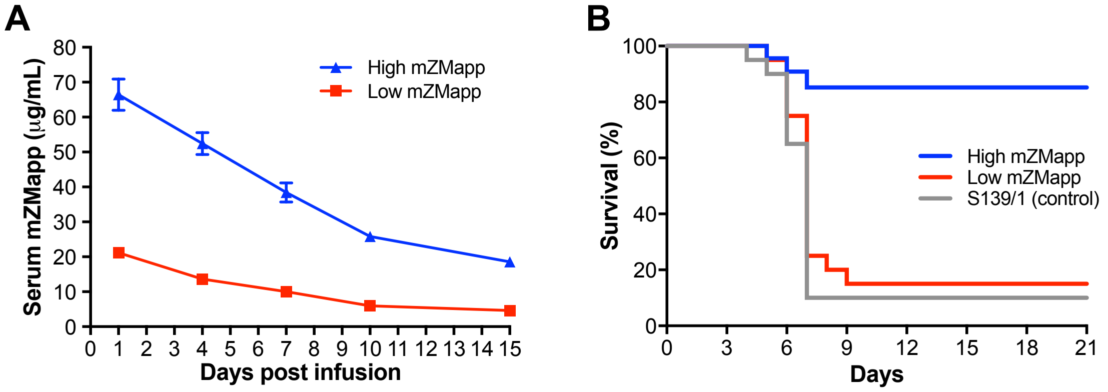

Figure S6

Passive infusion of high-dose mZMapp protects mice from Ebola challenge.

(A) The pharmacokinetic properties of mZMapp were initially evaluated in BALB/c mice. Mice (n=6/group) were infused via the intraperitoneal route with high dose mZMapp (40 µg/mAb; 120 µg total; ~6 mg/kg total) or low dose mZMapp (10 µg/mAb, 30 µg total; ~1.5 mg/kg total) on day 0. Blood was collected at the indicated time points and total mZMapp concentrations were evaluated by GP binding ELISA. Mean ± SEM are shown. **(B)** In a separate experiment, mice (n=20/group) were infused with a low or high dose of mZMapp or an anti-influenza mAb, S139/1 (30 µg total) as a control, one day prior to intraperitoneal challenge with 100 PFU mouse-adapted Ebola virus. Mice were monitored for lethality for 21 days. The Kaplan-Meier plot depicting survival is shown.

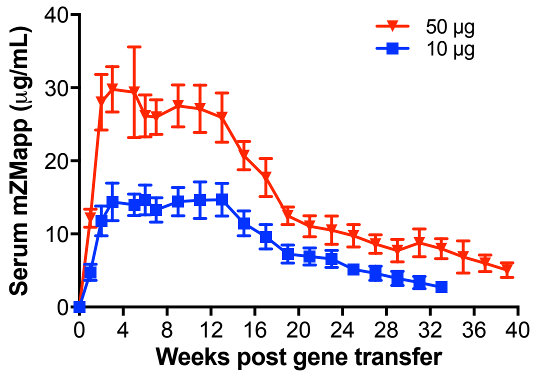

Figure S7

Duration of mZMapp expression following pDNA/EP administration.

BALB/c mice (n=6-9) were pretreated with 20 U of hyaluronidase and administered 10 or 50 µg H/L gWiz pDNA encoding each 13C6, 2G4 and 4G7 at distinct sites followed by EP. Total serum mAb concentrations were measured by GP-binding ELISAs. All data are represented as mean ± SEM.

Supplemental Methods

5A8 / 5A8-like Ab *in vitro* expression

5A8 constructs were evaluated for mAb expression by transient transfection in 293A cells using 5 µg/mL polyethylenimine and 1 µg pDNA. Transfected cells were maintained in Hybridoma-SFM medium for 72 hours. CD4 binding ELISA was used to quantify 5A8 in culture supernatants. Culture supernatants were collected, filtered, purified using Protein G Plus Agarose (Pierce). Eluted mAbs were concentrated by centrifugation (Millipore). mAbs were analyzed by SDS-PAGE visualized with Simply Blue and Western blot detected with anti-mouse HRP.

5A8 assessment for soluble CD4 binding in competition ELISA

In vitro produced 5A8 was assessed for its ability to compete with HRP-labeled iMab for soluble CD4 binding as previously described[1].

HIV-1 pseudovirus-based *in vitro* neutralization assays

Molecular cloned Env-pseudotyped virus G16 was prepared[2] and neutralization was assessed using TZM-bl cells (NIH AIDS Research and Reference Reagent Program, Division of AIDS, NIAID, NIH) with a luciferase-based assay as previously described[1].

Supplemental References

1. Seaman, MS, Janes, H, Hawkins, N, Grandpre, LE, Devoy, C, Giri, A*, et al.* (2010). Tiered categorization of a diverse panel of HIV-1 Env pseudoviruses for assessment of neutralizing antibodies. *Journal of virology* **84**: 1439-1452.
2. Sun, M, Pace, CS, Yao, X, Yu, F, Padte, NN, Huang, Y*, et al.* (2014). Rational design and characterization of the novel, broad and potent bispecific HIV-1 neutralizing antibody iMabm36. *J Acquir Immune Defic Syndr* **66**: 473-483.
