# Supplementary material for: In Vivo Production of Monoclonal Antibodies by Gene Transfer via Electroporation Protects against Lethal Influenza and Ebola Infections
Source: Mol Ther Methods Clin Dev. 2017 Sep 20;7:74–82. doi: 10.1016/j.omtm.2017.09.003 (PMC5633264; doi:10.1016/j.omtm.2017.09.003)
Supplement: Document S2. Article plus Supplemental Information [file mmc2.pdf]

Molecular Therapy
Methods & Clinical Development
Original Article

In Vivo Production of Monoclonal Antibodies by Gene Transfer via Electroporation Protects against Lethal Influenza and Ebola Infections

Chasity D. Andrews,[1,4] Yang Luo,[1,4] Ming Sun,[1,3,4] Jian Yu,[1] Arthur J. Goff,[2] Pamela J. Glass,[2] Neal N. Padte,[1] Yaoxing Huang,[1,5] and David D. Ho[1,5]

[1]Aaron Diamond AIDS Research Center, The Rockefeller University, New York, NY 10016, USA; [2]US Army Medical Research Institute of Infectious Diseases, Frederick, MD 21702, USA

Monoclonal antibodies (mAbs) have wide clinical utility, but global access is limited by high costs and impracticalities associated with repeated passive administration. Here, we describe an optimized electroporation-based DNA gene transfer platform technology that can be utilized for production of functional mAbs in vivo, with the potential to reduce costs and administration burdens. We demonstrate that multiple mAbs can be simultaneously expressed at protective concentrations for a protracted period of time using DNA doses and electroporation conditions that are feasible clinically. The expressed mAbs could also protect mice against lethal influenza or Ebola virus challenges. Our findings suggest that this DNA gene transfer platform technology could be a game-changing advance that expands access to effective mAb therapeutics globally.

INTRODUCTION

Monoclonal antibodies (mAbs) are among the fastest growing class of therapeutics being developed for a broad range of indications including cancer, inflammatory disorders, and infectious diseases.[1] Many mAb therapeutics like trastuzumab (Herceptin) cost as much as $100,000 annually per patient,[2] resulting in reduced access in many global markets. Due to the inherent high costs associated with antibody manufacturing facilities and processes, biosimilars will only marginally decrease the cost of mAb therapeutics. In addition, the mAb storage conditions and repeated administrations are impractical for many developing countries. A major technological breakthrough is therefore required to make mAb therapeutics available and affordable globally.

One potentially transformative approach to antibody therapy is to manufacture the mAb in the patient. Genes encoding the mAb could be introduced into certain host cells (e.g., muscle), which then serve as the “factory” for in vivo antibody production. Conceptually, such a gene transfer strategy has been demonstrated in animal models using viral vectors such as adeno-associated virus (AAV) providing antibody protection against respiratory syncytial virus (RSV),[3] simian immunodeficiency virus (SIV),[4] HIV type 1 (HIV-1),[5] and influenza viruses.[6–8] The near permanence of in vivo antibody production elicited by systemic AAV vector delivery renders this approach more similar to vaccination. Although intranasal delivery of AAV offers the potential to decrease the duration of expression,[6] the prolonged persistence of high-level mAb production with systemic AAV delivery raises concerns of adverse consequences that might manifest only months or years later, and this remains a major regulatory hurdle for systemic AAV-mediated antibody gene transfer.

Another approach to antibody gene transfer is to utilize plasmid DNA (pDNA). A vast number of vaccine candidates use pDNA to express antigens in vivo. pDNA has been tested for mAb production because pDNA is easy to manufacture, lacks cold-chain storage requirements, and has a favorable clinical safety profile to date.[9] The transgene transduction and expression are typically low after intramuscular (i.m.) injection, unless in vivo electroporation (EP) is applied concurrently. EP functions through the application of electric pulses resulting in cell membrane destabilization and DNA electrophoresis facilitating DNA delivery into cells.[10] Presumably, mAbs are expressed endogenously by transduced muscle cells and released into the circulation. Previous studies on pDNA/EP for antibody gene transfer have shown that mAbs produced in vivo are functionally intact and can protect mice from influenza,[11,12] Dengue,[13] or Chikungunya virus challenge.[14] Although some of these studies demonstrated persistence of appreciable in vivo antibody productions for weeks to months,[11–14] others did not.[15–17] Importantly, prior pDNA/EP efforts typically yielded low serum/plasma antibody concentrations (~<1 μg/mL)[13–16] while using doses of pDNA (25–300 μg) for a single antibody[11–17] that are too high to scale up for human use.

Received 11 July 2017; accepted 13 September 2017;
https://doi.org/10.1016/j.omtm.2017.09.003.

[3]Present address: Institute of Medical Biology, Chinese Academy of Medical Science and Peking Union Medical College, Kunming, Yunnan, People’s Republic of China
[4]These authors contributed equally to this work.
[5]These authors contributed equally to this work.
Correspondence: David D. Ho, Aaron Diamond AIDS Research Center, The Rockefeller University, New York, NY 10016, USA.
E-mail: dho@adarc.org

 © 2017 The American Society of Gene and Cell Therapy.
This is an open access article under the CC BY license (http://creativecommons.org/licenses/by/4.0/).

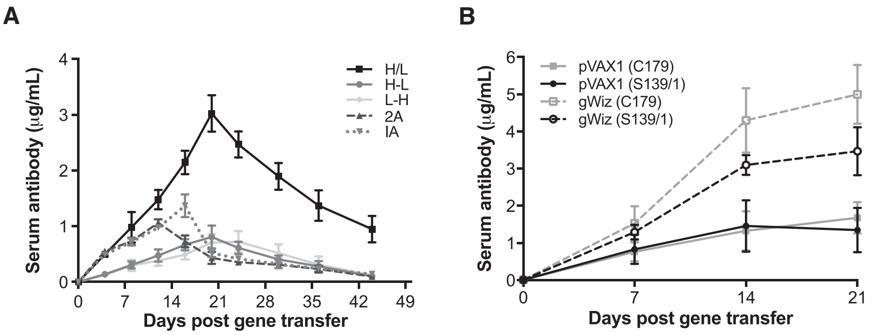

Figure 1. Optimized Gene Cassettes and Vector Backbone Enhance In Vivo mAb Expression

(A) Comparison of 5A8 mAb expression over time with different pDNA (50 μg) gene configurations in the pVAX1 vector administered by EP in BALB/c mice (n = 5–8). Serum 5A8 concentrations were measured by CD4 binding ELISAs. (B) gWiz expression vector yields higher mAb concentrations than pVAX1. Following 20 U of hyaluronidase pretreatment, BALB/c mice (n = 3) were administered 10 μg of pVAX1 (solid lines) or gWiz (dashed lines) encoding H/L of C179 or S139/1. Serum mAb concentrations were measured by HA-specific binding ELISAs. Data are presented as mean ± SEM. H/L, separate plasmids carrying H and L chain genes under CMV promoter; H-L, single plasmid with H- and L-chain genes under CMV and human elongation factor-1 alpha (hEF1α) promoters, respectively; L-H, single plasmid with L- and H-chain genes under CMV and hEF1α promoters, respectively; 2A, single plasmid with H- and L-chain genes under CMV promoter separated by furin cleavage site coupled with a P2A self-processing peptide; IA, immunoadhesin.

Here, we describe a systematic evaluation of pDNA/EP in order to place this platform technology for producing mAbs in vivo on the path for clinical development. Using clinically applicable experimental parameters, including EP conditions that are acceptable in humans and clinically feasible DNA doses, we can now achieve mAb concentrations in mice that are in the therapeutic range for a duration of several months. Moreover, we use this technology to express multiple mAbs in vivo simultaneously and demonstrate their protective efficacy against influenza and Ebola viruses, two of the greatest biothreats today.

RESULTS

Gene Cassette, Regimen, and Vector Optimizations Enhance mAb Expression

EP was previously shown to improve transgene expression of i.m. delivered pDNA.[15,16] We optimized gene transfer cassettes to minimize the amount of injected pDNA needed to obtain high mAb expression. Here, five gene cassette configurations utilizing the pVAX1 vector (Invitrogen Thermo Fisher Scientific, Grand Island, NY) were evaluated with 5A8, the mouse precursor mAb of an HIV-1 entry inhibitor ibalizumab (iMab)[18,19] as a model antibody (Figure S1A). Co-injection of separate plasmids carrying the heavy- (H) and light (L)-chain genes (H/L) under control of cytomegalovirus (CMV) promoter was compared with a single injection of dual-promoter plasmids containing H- and L-chain genes, as well as single-promoter plasmid constructs with the H- and L-chain genes separated by a furin cleavage site coupled with a P2A self-processing peptide (2A)[13,20] or the single-chain variable fragment (scFv) fused to the Fc region known as an immunoadhesin (IA) (Figure S1A).[4,6] All gene expression cassettes produced mAb or mAb-like molecules in vitro (Figure S1B) with binding and functional activities comparable with the clinical supply of iMab as assessed by ELISA and HIV-1 neutralization, respectively (Figure S1C). When compared following i.m. injection with EP in mice, co-injection of two plasmid gene cassettes (H/L) at a 1:1 ratio resulted in the highest serum mAb concentrations (Figure 1A) when normalized by the amount of pDNA injected. The serum 5A8 mAb produced by in vivo gene transfer with H/L plasmid gene cassettes was functional as demonstrated by ex vivo neutralization of HIV-1 pseudotyped virus (Figure S2A), and dose-dependent mAb expression was observed with pDNA administered via EP (Figure S2B).

The amount of pDNA used to generate such levels of functional mAb expression may be too high to translate to clinical application. In order to enhance transgene expression, pretreatment with hyaluronidase was added to the regimen. Hyaluronidase breaks down hyaluronan, a component of the skeletal muscle extracellular matrix, to facilitate DNA diffusion.[11,21,22] Consistent with previous reports,[11,12,21,23] 2.7- to 2.9-fold higher mAb expression was observed following hyaluronidase pretreatment (Figure S3). Next, we compared mAb expression with two commercially available DNA vaccine vectors, pVAX1 and gWiz (Aldevron, Fargo, ND, USA). Whereas pVAX1 (3.0 kb) is a small basic vector, gWiz (5.1 kb) is larger with the addition of an intron upstream to the transgene and extensive bacterial element orientation/composition optimization.[24] The H- and L-chain genes from two anti-influenza mAbs, C179[25] and S139/1,[26] were cloned into pVAX1 and gWiz. Mice were administered 10 μg H/L pDNA of an individual mAb followed by EP. At day 21, S139/1 and C179 concentrations were 2.6- and 3.0-fold higher, respectively, when the gWiz vector was used compared with the pVAX1 vector (Figure 1B). Therefore, gWiz was used as the expression vector for all subsequent studies.

Oligoclonal mAb Responses Induced by pDNA/EP Protect Mice from Influenza Infection

We ultimately aimed to test the prophylactic efficacy of the pDNA/EP mAb gene transfer approach in vivo using an influenza challenge model. To understand the impact of individual versus combination antibodies for influenza prevention, we first evaluated two H3-reactive mAbs, S139/1 and 9H10, for protection against influenza A/Aichi/2/68 (H3N2) challenge. Mouse anti-influenza mAbs were used to permit long-term studies in mice without the complication of cross-species anti-mAb responses. All mAbs were constructed utilizing immunoglobulin G2a (IgG2a), which preferentially interacts with Fc receptors for IgG (FcγRs),[27] a requirement for in vivo protection against influenza with broadly neutralizing anti-influenza

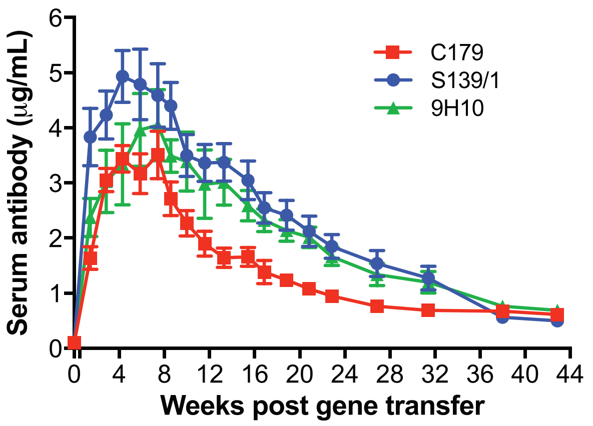

Figure 2. Optimized pDNA/EP Regimen Yields Durable Oligoclonal mAb Response in Mice

BALB/c mice (n = 8) were pretreated with 20 U of hyaluronidase and administered 10 µg of H/L gWiz pDNA encoding each C179, S139/1, and 9H10 at distinct sites followed by EP. Serum mAb concentrations were measured by HA-specific binding ELISAs. All data are presented as mean ± SEM.

mAbs.[27,28] Mice passively infused with 100 µg (~5 mg/kg) of either individual mAb 1 day prior to challenge were completely protected from influenza-related mortalities. Although 10 µg of individual mAb (~0.5 mg/kg) resulted in significant influenza-related morbidity and mortality, co-administering S139/1 and 9H10 at 10 µg/mAb enhanced the protective capacity of the mAbs resulting in complete protection from influenza-related mortalities, supporting the evaluation of an oligoclonal mAb response as influenza immunoprophylaxis (Figure S4).

To determine whether our gene transfer technology could express numerous mAbs simultaneously, we administered 10 µg of H/L pDNA in the gWiz backbone encoding for each of three anti-influenza mAbs—C179, a group 1 hemagglutinin-stalk-binding mAb,[25] S139/1, a broadly reactive group 1 and 2 head-binding mAb,[26] and 9H10, a group 2 hemagglutinin-stalk-binding mAb[29]—to mice at distinct sites followed by EP. The mAbs were selected such that two mAbs are reactive to both H1 viruses (C179 and S139/1) and H3 viruses (S139/1 and 9H10), the influenza subtypes primarily responsible for seasonal influenza infections, while targeting two different vulnerable sites on each virus. All three mAbs were durably expressed with peak serum mAb concentrations (3.5–4.9 µg/mL) achieved between weeks 4 and 8 with concentrations gradually decreasing to 0.5–0.7 µg/mL by week 43 (Figure 2).

Next, we evaluated the protective efficacy of an oligoclonal anti-influenza mAb response generated by pDNA/EP gene transfer against group 1 and 2 influenza challenges. BALB/c mice were injected i.m. in separate limbs with 10 µg of H/L pDNA in the gWiz backbone encoding each C179, S139/1, and 9H10 followed by EP. As controls, BALB/c mice were injected with 10 µg of H/L pDNA encoding each of three anti-Ebola murine mAbs (2G4, 4G7, and 13C6) followed by EP. Expression of individual anti-influenza mAbs was confirmed on days 12 and 19 after gene transfer, with mean serum concentrations of 2.0–6.2 µg/mL per mAb (Figures S5A and S5C) compared with mean total anti-Ebola mAb concentrations of 10.4–17.4 µg/mL (Figures S5B and S5D). Mice were challenged with 39 MLD_{50} (median lethal dose) of A/WSN/33 (H1N1) 23 days after mAb gene transfer. Weight loss in control animals was substantial beginning on day 2, whereas weight loss in mice expressing anti-influenza mAbs was less prominent (Figure 3A). Importantly, 9 of the 10 mice expressing anti-influenza mAbs survived the challenge compared with 0 of 8 mice expressing anti-Ebola mAbs ($p < 0.0001$, log rank test; Figure 3A). To examine the breadth of this gene transfer strategy, another set of mice treated with pDNA/EP was challenged as described above with 21 MLD_{50} of A/Aichi/2/68 (H3N2). Seven of 10 mice survived challenge, although mice expressing anti-influenza mAbs initially became sick as indicated by weight loss (Figure 3B). In comparison, the Ebola mAb-expressing mice became ill with greater rapidity and marked weight loss, resulting in protocol mandated humane sacrifice 4–6 days after challenge ($p < 0.0001$, log rank test; Figure 3B). These results demonstrate that an oligoclonal anti-influenza mAb response generated in vivo by gene transfer can protect from both group 1 (H1N1) and 2 (H3N2) influenza A strains.

To investigate whether the animals developed heterosubtypic immunity, we challenged the surviving mice from Figure 3 with the heterosubtypic virus 27.5 weeks after gene transfer of the anti-influenza mAb cocktail. Based on pharmacokinetic data from a similar study (Figure 2), the expressed serum mAb concentrations by pDNA/EP were expected to be low (0.7–1.5 µg/mL) but significant at the time of the heterosubtypic challenge. The seven mice protected from A/Aichi/2/68 (H3N2) were subsequently challenged with A/WSN/33 (H1N1; Figure 4A), whereas the nine mice protected from A/WSN/33 (H1N1) were subsequently challenged with A/Aichi/2/68 (H3N2) (Figure 4B). In both cases, 100% of previously challenged mice were protected from heterosubtypic challenge, with minimal weight loss and no mortality observed compared with control mice, all of which succumbed to infection (Figure 4). In fact, protection against mortality was more complete in the second challenge. These results suggest that oligoclonal immunoprophylaxis provided by pDNA/EP not only protects mice from viral challenge (Figure 3), but also most likely permits the generation of a host immune response to a heterologous influenza strain (Figure 4).

Oligoclonal mAb Responses Protect Mice from Ebola Virus Infection

To investigate the robustness of this gene delivery platform, we next applied pDNA/EP to the prevention of a different infectious disease where successful protection has been afforded by oligoclonal mAb responses. The 2014 Ebola virus epidemic in West Africa and the likelihood of future outbreaks highlight the critical need for rapid and effective prophylaxis and treatment options. ZMapp, an mAb cocktail consisting of 13C6, 2G4, and 4G7, is currently the only anti-Ebola mAb cocktail used in humans,[30] and it afforded complete protection in macaques when administered as post-exposure

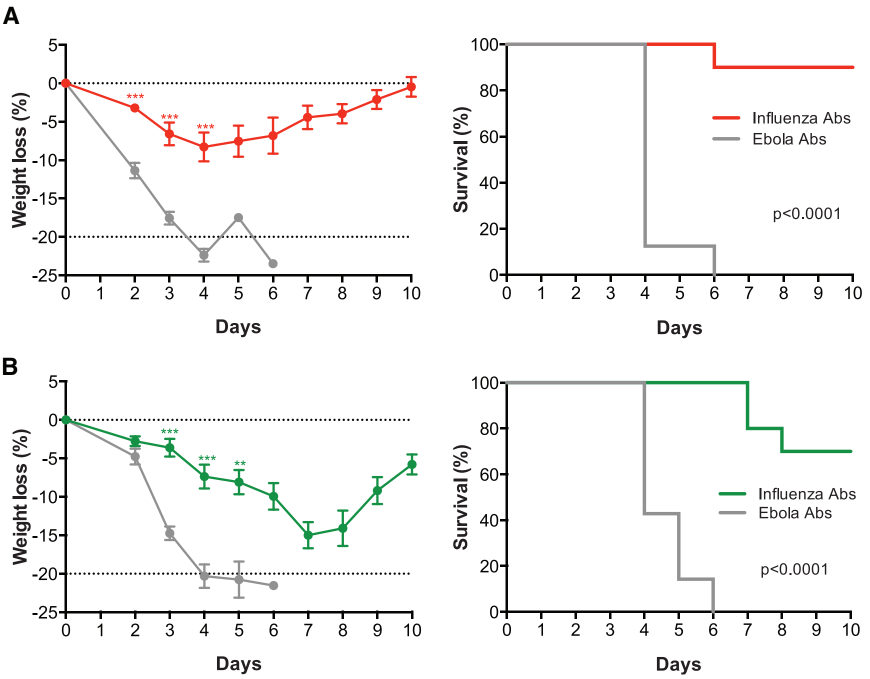

Figure 3. Oligoclonal mAb Response Protects Mice from Group 1 or Group 2 Influenza Challenges

BALB/c mice (n = 20) were pretreated with 20 U of hyaluronidase and administered 10 μg of H/L gWiz pDNA per anti-influenza mAb (C179, S139/1, and 9H10) in muscles of separate limbs followed by EP. For the control group, BALB/c mice (n = 15) were pretreated with 20 U of hyaluronidase and administered 10 μg of H/L gWiz pDNA per anti-Ebola mAb (2G4, 4G7, and 13C6) at distinct sites with EP. Blood was collected on days 12, 19, and 33 (in surviving mice) for pharmacokinetic analyses (Figure S5). (A and B) On day 23, the mice were randomized and anti-influenza DNA/EP mice (n = 10) and anti-Ebola DNA/EP mice (n = 7–8) were challenged with (A) 39 MLD_{50} of A/WSN/33 (H1N1) or (B) 21 MLD_{50} of A/Aichi/2/68 (H3N2) delivered intranasally. (A and B) Mean weight loss (%) ± SEM compared with day 0 (left) and survival (%) as depicted in Kaplan-Meier plots (right) following (A) H1N1 and (B) H3N2 challenge. For percent weight loss graphs, **p = 0.0021, ***p < 0.0001.

prophylaxis within 5 days of challenge.[31] ZMapp components have been tested individually or in other combinations as prophylaxis in mice by passive transfer,[32,33] whereas the ZMapp combination conferred protection when delivered as prophylaxis by AAV.[34] Here, we evaluated the prophylactic efficacy of ZMapp when delivered by pDNA/EP.

To avoid cross-species immunogenicity, we constructed the ZMapp antibodies with murine IgG2a Fc (mZMapp). Passively administered mZMapp at low (10 μg/mAb; 30 μg total) or high (40 μg/mAb; 120 μg total) doses resulted in mean serum mZMapp concentrations 1 day after injection of 21.2 and 66.4 μg/mL, respectively, and a normal half-life of 6.2–7.2 days (Figure S6A). In a follow-up experiment, mice were passively infused with the same mZMapp doses and challenged with 100 plaque-forming units (PFUs) of mouse-adapted Ebola virus (1976 strain, Mayinga[35]) 1 day after mAb administration. High protective efficacy (17/20 mice; 85%) was observed in mice administered 120 μg of mZMapp (~6 mg/kg total mAb; Figure S6B). However, no significant difference was observed between mice receiving 30 μg of mZMapp (~1.5 mg/kg total mAb) and control mice administered 30 μg of S139/1, an anti-influenza mAb serving as control (Figure S6B).

We then evaluated a range of pDNA doses (5–50 μg) encoding H/L of each of the three mZMapp mAbs in the gWiz vector in the gene transfer experiments. Serum mZMapp levels were generally pDNA dose dependent, with the highest or lowest levels observed in the group injected with 50 or 5 μg pDNA/mZMapp mAb, respectively. Minimal differences were observed between the groups of mice injected with 10 or 25 μg pDNA/mZMapp mAb (Figure 5A). Similar trends were observed 14 days after pDNA/EP administration (Figure 5B). Mice (n = 20 per group) were administered 5, 10, 25, or 50 μg of pDNA for each of the three mZMapp mAbs to evaluate the protective efficacy of this pDNA/EP regimen. As controls, mice (n = 30) were administered 150 μg of pDNA encoding H/L of the anti-influenza mAb, S139/1. Blood was collected 14 days after pDNA/EP administration, and the mean total serum mZMapp concentrations were 8.2, 10.7, 13.6, and 35.6 μg/mL, respectively (Figure 5B). The mice were subsequently challenged 28 or 31 days after pDNA/EP administration with 100 PFUs of a mouse-adapted Ebola virus (1976 strain, Mayinga[35]). Complete protection from death was observed in the mice administered 10 or 50 μg of pDNA/mZMapp mAb (Figure 5C). One mouse administered 25 μg of pDNA/mZMapp mAb succumbed to disease on day 7 after challenge, resulting in 95% (19/20 mice) protection. At a dose of 5 μg of pDNA/mZMapp mAb, 70% (14/20 mice) protection was noted. In contrast, all 30 control mice succumbed to infection 3–8 days after challenge (Figure 5C). Interestingly, all mice in the 5 μg pDNA group displayed signs of infection 4 days after challenge (Figure 5D). In comparison, 7 of 20 mice in the 10 μg pDNA group exhibited signs of illness on days 7–8, whereas only 3 of 20 mice in the 25 μg pDNA group exhibited disease signs (Figure 5D). Based on previous pharmacokinetic data (Figure 5A), the mean total serum mZMapp concentrations at week 5, near the time of challenge, were expected to be 8.8, 11.5, 13.4, and 29.4 μg/mL for the 5, 10, 25, and 50 μg pDNA groups, respectively, indicating a correlation between the total serum mZMapp concentration and protective efficacy. Sustained antibody expression for at least 15 weeks with a half-life of 9.5–12.2 weeks (66–85 days) was observed in mice administered 10 or 50 μg of pDNA/mZMapp mAb prior to a slow decrease in expression (Figure S7); therefore, it is expected that the protective duration provided by pDNA/EP administration might be at least 2–3 months.

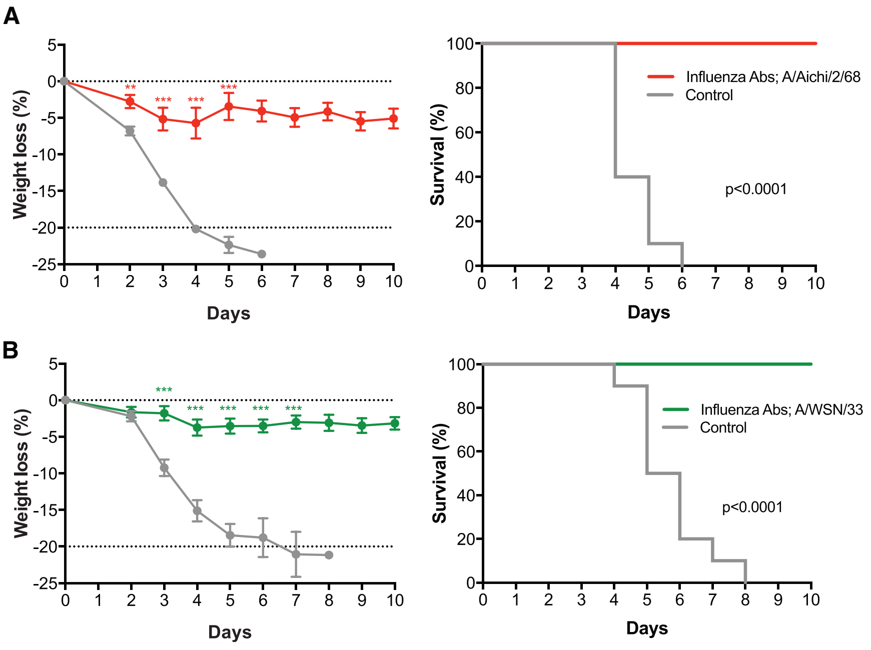

Figure 4. Heterosubtypic Immunity Protects Mice from Influenza Challenge

(A and B) BALB/c mice surviving the A/Aichi/2/68 challenge (n = 7) or A/WSN/33 challenge (n = 9) (from Figure 3) were subsequently challenged intranasally 27.5 weeks after the initial challenge with (A) 39 LD_{50} A/WSN/33 (H1N1) or (B) 21 LD_{50} of A/Aichi/2/68 (H3N2), respectively. Control mice were 12 weeks of age at the time of challenge. (Left) Mean weight loss (%) ± SEM compared with day 0 and (right) survival (%) as depicted in Kaplan-Meier plots following challenge. For percent weight loss graphs, **p = 0.0016, ***p ≤ 0.0001.

DISCUSSION

The current costs associated with mAb therapies, the practicality of passive infusion administration, and the necessity of repeated dosing prohibit global availability. We have aimed to pursue the production of mAb in vivo via gene transfer technologies that could be substantially cheaper and easier, thereby expanding access to antibody medicines in areas beyond the wealthier nations. Here, we report the results of our gene transfer experiments using pDNA delivered by EP to produce multiple functional mAbs in mice. We believe our in-depth studies have moved pDNA/EP beyond preliminary reports, placing this platform technology on a solid foundation for clinical development. For our efficacy experiments in mice, we used doses of pDNA (5–10 μg) that are scalable for humans, in contrast to previous studies (25–300 μg).[11–17] Despite lower inoculum of pDNA, we observed higher mAb expression with mean peak serum concentrations (3–5 μg/mL) (Figure 2) that are 3- to 10-fold higher than previous findings.[13–17] Furthermore, mAb concentrations remained ~1 μg/mL for up to 32 weeks (Figure 2), which is more persistent that previously described,[14,16,17] therefore potentially extending the therapeutic window for treatment and prevention. The observed serum antibody concentrations fall well within the therapeutic range for many mAbs in clinical use today,[1,36] and the duration of antibody expression bodes well for infrequent administration of pDNA/EP. It is worth noting that in our studies, animals were challenged ~3–4 weeks after DNA/EP administration, implying the durability of protection and potentially impacting disease prevention in developing regions.

We demonstrate that our pDNA/EP gene transfer platform can produce functional mAbs in vivo against viral pathogens that are among the greatest biothreats to humanity today. Specifically, the expressed mAbs conferred solid protection against parenteral challenges with influenza viruses (groups 1 and 2) or a strain of Ebola virus. We note that we achieved protection using weakly neutralizing first generation mAbs. Much more potent mAbs directed against influenza and Ebola viruses have been developed,[37–44] and their protective efficacies are expected to be even more superior when delivered by pDNA/EP. We also note that our current study demonstrates simultaneous expression of multiple mAbs in vivo, which may be crucial in affording protection because it has the potential of providing greater activity and/or breadth.

Multiple gene transfer approaches are being pursued to express therapeutic mAbs in vivo. AAV delivery of mAb genes into muscle can yield high levels of antibody expression that continues for years.[4,45] However, the near permanence of antibody expression poses significant regulatory concerns that must be overcome, unless an effective "off switch" could be engineered. Delivery of gene-encoding RNA could also result in the production of a protein or mAb in vivo.[46] Therapeutic serum concentrations could be attained acutely, but the translational half-life is typically in the order of hours to a day,[47] yielding a pharmacokinetic profile similar to the passive infusion of the therapeutic protein or mAb at best.[46] Comparing and contrasting these gene transfer approaches with our findings using pDNA/EP, the current "sweet spot" of potential clinical use appears to be years for AAV, months for DNA, and days for RNA delivery strategies. Antibody expression durations of several months can be ideal for situations such as seasonal outbreaks when permanent expression is unnecessary, providing a therapeutic window sufficient to impact when global prevention access is limited. Additionally, the development of heterosubtypic immunity during antibody expression has broad implications for protection against diverse influenza strains.

The EP device we utilized here (Ichor's TriGrid Delivery System[48]), along with the electric field parameters, not only boosted the immunogenicity of a DNA vaccine, but also demonstrated an acceptable safety, tolerability, and acceptance profile in humans.[49] One challenge we face in moving this pDNA/EP technology into human use involves

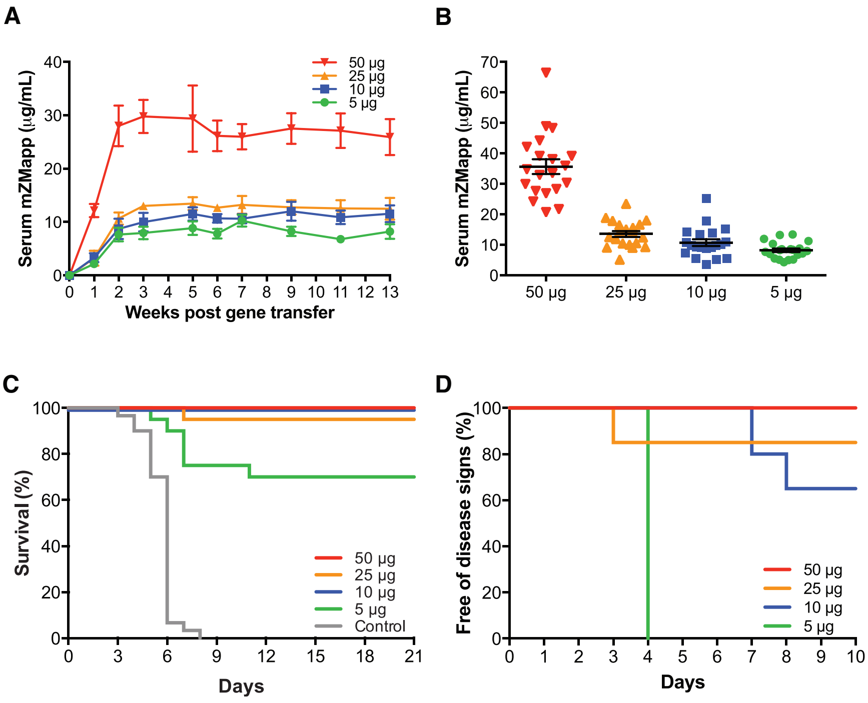

Figure 5. mZMapp Delivered by pDNA/EP Protects Mice from Lethal Ebola Virus Challenge

(A) BALB/c mice (n = 6–9) were pretreated with 20 U of hyaluronidase and administered 5, 10, 25, or 50 μg H/L gWiz pDNA encoding each 2G4, 4G7, and 13C6 in muscles of separate limbs followed by EP. Total serum mZMapp concentrations were measured by GP binding ELISAs. All data are presented as mean ± SEM. (B–D) BALB/c mice (n = 20/group) were pretreated with 20 U of hyaluronidase and administered 5, 10, 25, or 50 μg of H/L gWiz pDNA per Ebola mAb that comprised the mZMapp cocktail (2G4, 4G7, and 13C6) or 150 μg of H/L gWiz pDNA of S139/1 as a control. (B) Blood was collected 14 days after pDNA/EP and analyzed for total mZMapp concentration by ELISA. Each point represents the value from an individual mouse. Error bars indicate mean ± SEM. (C) The mice were challenged 28–31 days after pDNA/EP administration with 100 PFU of mouse-adapted Ebola virus and monitored for lethality for 21 days. The Kaplan-Meier plot depicting survival is shown. Significance ($p < 0.0001$, log rank test) is reported for each group electroporated with DNA encoding mZMapp compared with the control group. (D) The mice were monitored daily for onset of signs of disease following challenge, and the Kaplan-Meier plot depicting animals remaining free of disease signs is shown.

scaling from mice to humans, which may be addressed in part by engineering mAbs with improved pharmacokinetics and plasmid vectors with enhanced expression.[50] In addition, it is important to note that EP devices and conditions in clinical use today were designed years ago. Recent advances made in the field of EP in vivo will likely improve the expression level.[51] Furthermore, to date, EP has largely been applied to DNA vaccination, where a certain degree of cell death and inflammation is beneficial to the generation of an immune response. On the other hand, persistent and prolonged mAb production in vivo demands EP parameters that minimize cell death and immune recognition. We therefore firmly believe there is a lot of room for EP optimization (electric field settings and electrode arrays) that could overcome the challenges in the mouse-to-human scale-up. If successful, gene transfer via pDNA/EP could become a transformative platform technology that lowers the cost of and expands access to mAb therapy worldwide.

MATERIALS AND METHODS

Plasmid Construction

All 5A8 expression cassettes were separately constructed by PCR amplification and separately cloned into the pVAX1 expression plasmid at the *Nhe*I and *Xho*I restriction sites. IgG2a H- and L-chain DNA encoding C179,[25] S139/1 (GenBank: 4GMT_I and 4GMT_M), 9H10 (kindly provided by Dr. Peter Palese), 2G4 (patent US20120283414), 4G7 (patent US20120283414), and 13C6 (patent US6875433 B2) were optimized and synthesized (GeneArt Gene Synthesis, Thermo Fisher Scientific, Waltham, MA, USA) and cloned separately into the pVAX1 or gWiz expression plasmids. pDNA was isolated using EndoFree Plasmid Maxi kits (QIAGEN, Valencia, CA, USA). DNA yield and quality were confirmed by spectrophotometry and agarose gel electrophoresis.

Influenza Virus Production and Quantification

The mouse-adapted A/WSN/33 strain was obtained by passaging the virus seven times in BALB/c mice as previously described.[52] The MLD_{50} was determined in BALB/c mice. Mouse-adapted, BALB/c-titrated A/Aichi/2/68 was prepared as previously described.[53]

Mice, EP, and Virus Challenges

Animal Ethics Statement

Animals were maintained at the Comparative Bioscience Center of The Rockefeller University or at the US Army Medical Research Institute of Infectious Diseases (USAMRIID) facility in accordance with the regulations of the Institutional Animal Care and Use Committee (IACUC) of the housing institute. All animal studies were conducted under protocols approved by the IACUC of The Rockefeller University or the USAMRIID in compliance with the Animal Welfare Act and other federal statutes and regulations relating to animals and experiments involving animals.

pDNA/EP Administration

Six- to ten-week-old female BALB/c mice (Charles Rivers Laboratories) were administered pDNA by i.m. injection with EP using the TriGrid Delivery System (Ichor Medical Systems, San Diego, CA, USA) using conditions previously described.[48] Briefly, the electrode array consisted of an array of four penetrating needle-type electrodes 4 mm in length arranged in two interlocking equilateral triangles to form a diamond shape with an intraelectrode spacing of

2.5 mm. The electrode array included a central injection port designed to interface with a 0.3 cc insulin syringe and 30G injection needle (Becton Dickinson, Franklin Lakes, NY, USA). During administration, the electrode array and injection needle were inserted into the target administration site with the major axis of the array aligned with the orientation of the muscle fibers at the injection site. Following i.m. injection of the DNA, electrical stimulation was applied via the surrounding array of electrodes at an amplitude of 62.5 V (250 V/cm of electrode spacing) for a duration of 40 ms applied over a period of 400 ms (a 10% duty cycle). For hyaluronidase pretreatment, mice were injected i.m. with bovine hyaluronidase (Sigma, St. Louis, MO, USA) or PBS 2 hr prior to i.m. injection of pDNA with EP. For oligoclonal experiments, pDNA encoding H- and L-chains of one antibody were mixed, and pDNA encoding each antibody was injected in muscles in separate limbs to yield authentic antibodies. Mice were bled retroorbitally, and serum was isolated and frozen until analysis.

Influenza Prophylaxis in Mice

Influenza virus was thawed and diluted in PBS to deliver the indicated dose in 30 μL. Mice were anesthetized by isoflurane inhalation, and 15 μL of diluted virus was instilled into each nostril. Mice were weighed daily for 10 days after challenge and sacrificed when weight loss was >20% of starting weight as per IACUC regulations. Animal numbers per group were calculated to obtain statistical difference between no survival in the control group and at least 70% effectiveness in the treated groups with >90% power using a two-sided alpha level of 0.05.

Ebola Virus Prophylaxis in Mice

Mice were housed at the Comparative Bioscience Center of The Rockefeller University and administered pDNA/EP as described. Treated mice were then transported to the Biosafety Level 4 containment animal facility at USAMRIID, where the thoroughly validated lethal mouse-adapted Ebola virus mouse model was developed.[54] The mice were challenged intraperitoneally with 100 PFU of mouse-adapted Ebola virus (1976 strain, Mayinga[35]) and monitored daily for 21 days postinfection. Animal numbers per group (n = 20) were calculated to obtain statistical difference between 10% survival in the control group and at least 50% effectiveness in the treated groups with >90% power using a two-sided alpha level of 0.05.

ELISAs

Detection of 5A8 by sCD4 Binding

Plates coated with human soluble CD4 were blocked and incubated with mouse sera for 1 hr. Binding was detected using horseradish peroxidase (HRP)-conjugated goat anti-mouse IgG1 H&L (Bethyl Laboratories, Montgomery, TX, USA) or alkaline phosphatase (AP)-conjugated anti-mouse antibodies, and developed by 3,3′,5,5′-tetramethylbenzidine (TMB) or AMPAK kit (DAKO; Carpinteria, CA, USA). mAb concentration was determined by comparing absorbance values with the CD4 binding curve of purified 5A8 protein.

Detection of Mouse Anti-Influenza mAbs C179, S139/1, and 9H10

For co-expression studies, to discriminate the expression of S139/1 and 9H10, both recognizing H3 epitopes, S139/1 was detected using the HA1 subunit and 9H10 was detected with H10N8. ELISA plates coated with 200 ng of H1N1 (A/WSN/33) HA, 50 ng of H3N2 (A/Aichi/2/1968) HA1 or HA subunit, or 200 ng of H10N8 (A/Jiangxi-Donghu/346/2013) HA (Sino Biologicals, Beijing, China) were blocked and incubated with serially diluted mouse serum. After washing, goat anti-mouse IgG HRP was added for 1 hr at 37°C. Bound mAb was detected using TMB Liquid Substrate (Sigma) and stopped with 1N H_2SO_4. Spectrophotometric readings were performed at 450 nm with a 570 nm reference subtraction. Purified proteins were used for the standard curve.

Detection of Mouse Anti-Ebola mAbs 2G4, 4G7, and 13C6

ELISA plates were coated with 100 ng of Zaire GPΔTM in 0.1 M $NaHCO_3$ (pH 9.6) per well overnight at 4°C. Plates were washed and blocked with 5% milk and 0.5% BSA in PBS containing 0.05% Tween 20. Mouse sera were serially diluted in blocking buffer and incubated on the ELISA plate for 1 hr at 37°C. After washing, goat anti-mouse IgG HRP (Enzo Life Sciences, Plymouth Meeting, PA, USA) was added for 1 hr at 37°C. Bound mAb was detected using TMB Liquid Substrate (Sigma) and stopped with 1N H_2SO_4. Spectrophotometric readings were performed at 450 nm with a 570 nm reference subtraction. Purified proteins were used for the standard curve.

Statistical Methods

All statistical comparisons were performed using GraphPad Prism Software, version 6 (La Jolla, CA, USA). An unpaired two-tailed t test was used to assess weight loss differences in surviving mice. Survival differences were analyzed using the Mantel-Cox log rank test with the Bonferroni correction performed for multiple comparisons.

SUPPLEMENTAL INFORMATION

Supplemental Information includes Supplemental Materials and Methods and seven figures and can be found with this article online at https://doi.org/10.1016/j.omtm.2017.09.003.

AUTHOR CONTRIBUTIONS

C.D.A., Y.L., M.S., J.Y., A.J.G., P.J.G., and Y.H. performed experiments and data analysis. C.D.A., Y.L., A.J.G., P.J.G., N.N.P., Y.H., and D.D.H. conceived the study and designed experiments. C.D.A., Y.H., and D.D.H. wrote the manuscript with comments from all authors.

CONFLICTS OF INTEREST

C.D.A., J.Y., N.N.P., Y.H., and D.D.H. were consultants for RenBio, Inc., a company developing gene transfer platform technologies, and Y.H. and D.D.H. are co-founders of RenBio, Inc.

ACKNOWLEDGMENTS

We thank Mar Boente-Carrera, Mili R. Gajjar, and Lily Tsai for technical assistance with experiments and members of the Ho laboratory for helpful discussions. We thank Joshua Shamblin, Suzanne Wollen, Adrienne Kimmel, and Ashley Piper for technical assistance for in vivo Ebola challenge studies. We thank Ichor Medical Systems for kindly providing the electroporation device, Dr. Peter Palese for kindly providing the A/WSN/33 virus and 9H10 antibody sequences, and Drs. Richard W. Compans and Ioanna Skountzou for kindly providing the A/Aichi/2/68 virus. D.D.H. was supported by the Bill and Melinda Gates Foundation's Collaboration for AIDS Vaccine Discovery (grant OPP1040731), the Defense Advanced Research Projects Agency (contract W31PQ4-14-1-0010), and the Army Research Office through a subcontract of contract W911NF-14-C-0001 that was awarded in connection to the Defense Advanced Research Projects Agency's ADEPT PROTECT program. These findings do not necessarily reflect the position or policy of the Government, and no official endorsement should be inferred.

REFERENCES

1. Ecker, D.M., Jones, S.D., and Levine, H.L. (2015). The therapeutic monoclonal antibody market. MAbs *7*, 9–14.
2. Blackstone, E.A., and Joseph, P.F. (2013). The economics of biosimilars. Am. Health Drug Benefits *6*, 469–478.
3. Skaricic, D., Traube, C., De, B., Joh, J., Boyer, J., Crystal, R.G., and Worgall, S. (2008). Genetic delivery of an anti-RSV antibody to protect against pulmonary infection with RSV. Virology *378*, 79–85.
4. Johnson, P.R., Schnepp, B.C., Zhang, J., Connell, M.J., Greene, S.M., Yuste, E., Desrosiers, R.C., and Clark, K.R. (2009). Vector-mediated gene transfer engenders long-lived neutralizing activity and protection against SIV infection in monkeys. Nat. Med. *15*, 901–906.
5. Balazs, A.B., Chen, J., Hong, C.M., Rao, D.S., Yang, L., and Baltimore, D. (2011). Antibody-based protection against HIV infection by vectored immunoprophylaxis. Nature *481*, 81–84.
6. Limberis, M.P., Adam, V.S., Wong, G., Gren, J., Kobasa, D., Ross, T.M., Kobinger, G.P., Tretiakova, A., and Wilson, J.M. (2013). Intranasal antibody gene transfer in mice and ferrets elicits broad protection against pandemic influenza. Sci. Transl. Med. *5*, 187ra72.
7. Limberis, M.P., Racine, T., Kobasa, D., Li, Y., Gao, G.F., Kobinger, G., and Wilson, J.M. (2013). Vectored expression of the broadly neutralizing antibody FI6 in mouse airway provides partial protection against a new avian influenza A virus, H7N9. Clin. Vaccine Immunol. *20*, 1836–1837.
8. Balazs, A.B., Bloom, J.D., Hong, C.M., Rao, D.S., and Baltimore, D. (2013). Broad protection against influenza infection by vectored immunoprophylaxis in mice. Nat. Biotechnol. *31*, 647–652.
9. Ferraro, B., Morrow, M.P., Hutnick, N.A., Shin, T.H., Lucke, C.E., and Weiner, D.B. (2011). Clinical applications of DNA vaccines: current progress. Clin. Infect. Dis. *53*, 296–302.
10. Satkauskas, S., André, F., Bureau, M.F., Scherman, D., Miklavcic, D., and Mir, L.M. (2005). Electrophoretic component of electric pulses determines the efficacy of in vivo DNA electrotransfer. Hum. Gene Ther. *16*, 1194–1201.
11. Yamazaki, T., Nagashima, M., Ninomiya, D., Arai, Y., Teshima, Y., Fujimoto, A., Ainai, A., Hasegawa, H., and Chiba, J. (2011). Passive immune-prophylaxis against influenza virus infection by the expression of neutralizing anti-hemagglutinin monoclonal antibodies from plasmids. Jpn. J. Infect. Dis. *64*, 40–49.
12. Elliott, S.T.C., Kallewaard, N.L., Benjamin, E., Wachter-Rosati, L., McAuliffe, J.M., Patel, A., et al. (2017). DMAb inoculation of synthetic cross reactive antibodies protects against lethal influenza A and B infections. npj Vaccines *2*, 18.
13. Flingai, S., Plummer, E.M., Patel, A., Shresta, S., Mendoza, J.M., Broderick, K.E., Sardesai, N.Y., Muthumani, K., and Weiner, D.B. (2015). Protection against dengue disease by synthetic nucleic acid antibody prophylaxis/immunotherapy. Sci. Rep. *5*, 12616.
14. Muthumani, K., Block, P., Flingai, S., Muruganantham, N., Chaaithanya, I.K., Tingey, C., Wise, M., Reuschel, E.L., Chung, C., Muthumani, A., et al. (2016). Rapid and long-term immunity elicited by DNA-encoded antibody prophylaxis and DNA vaccination against Chikungunya virus. J. Infect. Dis. *214*, 369–378.
15. Perez, N., Bigey, P., Scherman, D., Danos, O., Piechaczyk, M., and Pelegrin, M. (2004). Regulatable systemic production of monoclonal antibodies by in vivo muscle electroporation. Genet. Vaccines Ther. *2*, 2.
16. Tjelle, T.E., Corthay, A., Lunde, E., Sandlie, I., Michaelsen, T.E., Mathiesen, I., and Bogen, B. (2004). Monoclonal antibodies produced by muscle after plasmid injection and electroporation. Mol. Ther. *9*, 328–336.
17. Muthumani, K., Flingai, S., Wise, M., Tingey, C., Ugen, K.E., and Weiner, D.B. (2013). Optimized and enhanced DNA plasmid vector based in vivo construction of a neutralizing anti-HIV-1 envelope glycoprotein Fab. Hum. Vaccin. Immunother. *9*, 2253–2262.
18. Kuritzkes, D.R., Jacobson, J., Powderly, W.G., Godofsky, E., DeJesus, E., Haas, F., Reimann, K.A., Larson, J.L., Yarbough, P.O., Curt, V., and Shanahan, W.R., Jr. (2004). Antiretroviral activity of the anti-CD4 monoclonal antibody TNX-355 in patients infected with HIV type 1. J. Infect. Dis. *189*, 286–291.
19. Dimitrov, A. (2007). Ibalizumab, a CD4-specific mAb to inhibit HIV-1 infection. Curr. Opin. Investig. Drugs *8*, 653–661.
20. Fang, J., Qian, J.J., Yi, S., Harding, T.C., Tu, G.H., VanRoey, M., and Jooss, K. (2005). Stable antibody expression at therapeutic levels using the 2A peptide. Nat. Biotechnol. *23*, 584–590.
21. McMahon, J.M., Signori, E., Wells, K.E., Fazio, V.M., and Wells, D.J. (2001). Optimisation of electrotransfer of plasmid into skeletal muscle by pretreatment with hyaluronidase—increased expression with reduced muscle damage. Gene Ther. *8*, 1264–1270.
22. Schertzer, J.D., Plant, D.R., and Lynch, G.S. (2006). Optimizing plasmid-based gene transfer for investigating skeletal muscle structure and function. Mol. Ther. *13*, 795–803.
23. Mennuni, C., Calvaruso, F., Zampaglione, I., Rizzuto, G., Rinaudo, D., Dammassa, E., Ciliberto, G., Fattori, E., and La Monica, N. (2002). Hyaluronidase increases electrogene transfer efficiency in skeletal muscle. Hum. Gene Ther. *13*, 355–365.
24. Williams, J.A. (2013). Vector design for improved DNA vaccine efficacy, safety and production. Vaccines (Basel) *1*, 225–249.
25. Okuno, Y., Isegawa, Y., Sasao, F., and Ueda, S. (1993). A common neutralizing epitope conserved between the hemagglutinins of influenza A virus H1 and H2 strains. J. Virol. *67*, 2552–2558.
26. Yoshida, R., Igarashi, M., Ozaki, H., Kishida, N., Tomabechi, D., Kida, H., Ito, K., and Takada, A. (2009). Cross-protective potential of a novel monoclonal antibody directed against antigenic site B of the hemagglutinin of influenza A viruses. PLoS Pathog. *5*, e1000350.
27. DiLillo, D.J., Tan, G.S., Palese, P., and Ravetch, J.V. (2014). Broadly neutralizing hemagglutinin stalk-specific antibodies require FcγR interactions for protection against influenza virus in vivo. Nat. Med. *20*, 143–151.
28. DiLillo, D.J., Palese, P., Wilson, P.C., and Ravetch, J.V. (2016). Broadly neutralizing anti-influenza antibodies require Fc receptor engagement for in vivo protection. J. Clin. Invest. *126*, 605–610.
29. Tan, G.S., Lee, P.S., Hoffman, R.M., Mazel-Sanchez, B., Krammer, F., Leon, P.E., Ward, A.B., Wilson, I.A., and Palese, P. (2014). Characterization of a broadly neutralizing monoclonal antibody that targets the fusion domain of group 2 influenza A virus hemagglutinin. J. Virol. *88*, 13580–13592.
30. Wec, A.Z., Herbert, A.S., Murin, C.D., Nyakatura, E.K., Abelson, D.M., Fels, J.M., He, S., James, R.M., de La Vega, M.A., Zhu, W., et al. (2017). Antibodies from a human survivor define sites of vulnerability for broad protection against Ebolaviruses. Cell *169*, 878–890.e815.

31. Qiu, X., Wong, G., Audet, J., Bello, A., Fernando, L., Alimonti, J.B., Fausther-Bovendo, H., Wei, H., Aviles, J., Hiatt, E., et al. (2014). Reversion of advanced Ebola virus disease in nonhuman primates with ZMapp. Nature *514*, 47–53.

32. Wilson, J.A., Hevey, M., Bakken, R., Guest, S., Bray, M., Schmaljohn, A.L., and Hart, M.K. (2000). Epitopes involved in antibody-mediated protection from Ebola virus. Science *287*, 1664–1666.

33. Qiu, X., Fernando, L., Melito, P.L., Audet, J., Feldmann, H., Kobinger, G., Alimonti, J.B., and Jones, S.M. (2012). Ebola GP-specific monoclonal antibodies protect mice and guinea pigs from lethal Ebola virus infection. PLoS Negl. Trop. Dis. *6*, e1575.

34. Limberis, M.P., Tretiakova, A., Nambiar, K., Wong, G., Racine, T., Crosariol, M., Xiangguo, Q., Kobinger, G., and Wilson, J.M. (2016). Adeno-associated virus serotype 9-expressed ZMapp in mice confers protection against systemic and airway-acquired Ebola virus infection. J. Infect. Dis. *214*, 1975–1979.

35. Bray, M., Davis, K., Geisbert, T., Schmaljohn, C., and Huggins, J. (1999). A mouse model for evaluation of prophylaxis and therapy of Ebola hemorrhagic fever. J. Infect. Dis. *179* (*Suppl 1*), S248–S258.

36. Ho, R.J.Y., and Gibaldi, M. (2013). Antibodies and derivatives. In Biotechnology and Biopharmaceuticals: Transforming Proteins and Genes into Drugs, Second Edition, R.J.Y. Ho, ed. (Wiley-Blackwell), pp. 139–210.

37. Throsby, M., van den Brink, E., Jongeneelen, M., Poon, L.L., Alard, P., Cornelissen, L., Bakker, A., Cox, F., van Deventer, E., Guan, Y., et al. (2008). Heterosubtypic neutralizing monoclonal antibodies cross-protective against H5N1 and H1N1 recovered from human IgM+ memory B cells. PLoS ONE *3*, e3942.

38. Corti, D., Voss, J., Gamblin, S.J., Codoni, G., Macagno, A., Jarrossay, D., Vachieri, S.G., Pinna, D., Minola, A., Vanzetta, F., et al. (2011). A neutralizing antibody selected from plasma cells that binds to group 1 and group 2 influenza A hemagglutinins. Science *333*, 850–856.

39. Bornholdt, Z.A., Turner, H.L., Murin, C.D., Li, W., Sok, D., Souders, C.A., Piper, A.E., Goff, A., Shamblin, J.D., Wollen, S.E., et al. (2016). Isolation of potent neutralizing antibodies from a survivor of the 2014 Ebola virus outbreak. Science *351*, 1078–1083.

40. Wang, W., Sun, X., Li, Y., Su, J., Ling, Z., Zhang, T., Wang, F., Zhang, H., Chen, H., Ding, J., and Sun, B. (2016). Human antibody 3E1 targets the HA stem region of H1N1 and H5N6 influenza A viruses. Nat. Commun. *7*, 13577.

41. Corti, D., Misasi, J., Mulangu, S., Stanley, D.A., Kanekiyo, M., Wollen, S., Ploquin, A., Doria-Rose, N.A., Staupe, R.P., Bailey, M., et al. (2016). Protective monotherapy against lethal Ebola virus infection by a potently neutralizing antibody. Science *351*, 1339–1342.

42. Misasi, J., Gilman, M.S., Kanekiyo, M., Gui, M., Cagigi, A., Mulangu, S., Corti, D., Ledgerwood, J.E., Lanzavecchia, A., Cunningham, J., et al. (2016). Structural and molecular basis for Ebola virus neutralization by protective human antibodies. Science *351*, 1343–1346.

43. Dreyfus, C., Laursen, N.S., Kwaks, T., Zuijdgeest, D., Khayat, R., Ekiert, D.C., Lee, J.H., Metlagel, Z., Bujny, M.V., Jongeneelen, M., et al. (2012). Highly conserved protective epitopes on influenza B viruses. Science *337*, 1343–1348.

44. Flyak, A.I., Shen, X., Murin, C.D., Turner, H.L., David, J.A., Fusco, M.L., Lampley, R., Kose, N., Ilinykh, P.A., Kuzmina, N., et al. (2016). Cross-reactive and potent neutralizing antibody responses in human survivors of natural Ebolavirus infection. Cell *164*, 392–405.

45. Balazs, A.B., and West, A.P., Jr. (2013). Antibody gene transfer for HIV immunoprophylaxis. Nat. Immunol. *14*, 1–5.

46. Sahin, U., Karikó, K., and Türeci, Ö. (2014). mRNA-based therapeutics—developing a new class of drugs. Nat. Rev. Drug Discov. *13*, 759–780.

47. Pardi, N., Tuyishime, S., Muramatsu, H., Kariko, K., Mui, B.L., Tam, Y.K., Madden, T.D., Hope, M.J., and Weissman, D. (2015). Expression kinetics of nucleoside-modified mRNA delivered in lipid nanoparticles to mice by various routes. J. Control. Release *217*, 345–351.

48. Hannaman, D. (2012). Electroporation based Trigrid^{TM} Delivery System (TDS) for DNA vaccine administration. In Gene Vaccines, J. Thalhamer, R. Weiss, and S. Scheiblhofer, eds. (Springer).

49. Vasan, S., Hurley, A., Schlesinger, S.J., Hannaman, D., Gardiner, D.F., Dugin, D.P., Boente-Carrera, M., Vittorino, R., Caskey, M., Andersen, J., et al. (2011). In vivo electroporation enhances the immunogenicity of an HIV-1 DNA vaccine candidate in healthy volunteers. PLoS ONE *6*, e19252.

50. Kay, M.A., He, C.Y., and Chen, Z.Y. (2010). A robust system for production of minicircle DNA vectors. Nat. Biotechnol. *28*, 1287–1289.

51. Rosazza, C., Meglic, S.H., Zumbusch, A., Rols, M.P., and Miklavcic, D. (2016). Gene electrotransfer: a mechanistic perspective. Curr. Gene Ther. *16*, 98–129.

52. Cottey, R., Rowe, C.A., and Bender, B.S. (2001). Influenza virus. Curr. Protoc. Immunol. *Chapter 19*. Unit 19.11.

53. Koutsonanos, D.G., del Pilar Martin, M., Zarnitsyn, V.G., Sullivan, S.P., Compans, R.W., Prausnitz, M.R., and Skountzou, I. (2009). Transdermal influenza immunization with vaccine-coated microneedle arrays. PLoS ONE *4*, e4773.

54. Gibb, T.R., Bray, M., Geisbert, T.W., Steele, K.E., Kell, W.M., Davis, K.J., and Jaax, N.K. (2001). Pathogenesis of experimental Ebola Zaire virus infection in BALB/c mice. J. Comp. Pathol. *125*, 233–242.

OMTM, Volume 7

Supplemental Information

In Vivo Production of Monoclonal Antibodies by Gene Transfer via Electroporation Protects against Lethal Influenza and Ebola Infections

Chasity D. Andrews, Yang Luo, Ming Sun, Jian Yu, Arthur J. Goff, Pamela J. Glass, Neal N. Padte, Yaoxing Huang, and David D. Ho

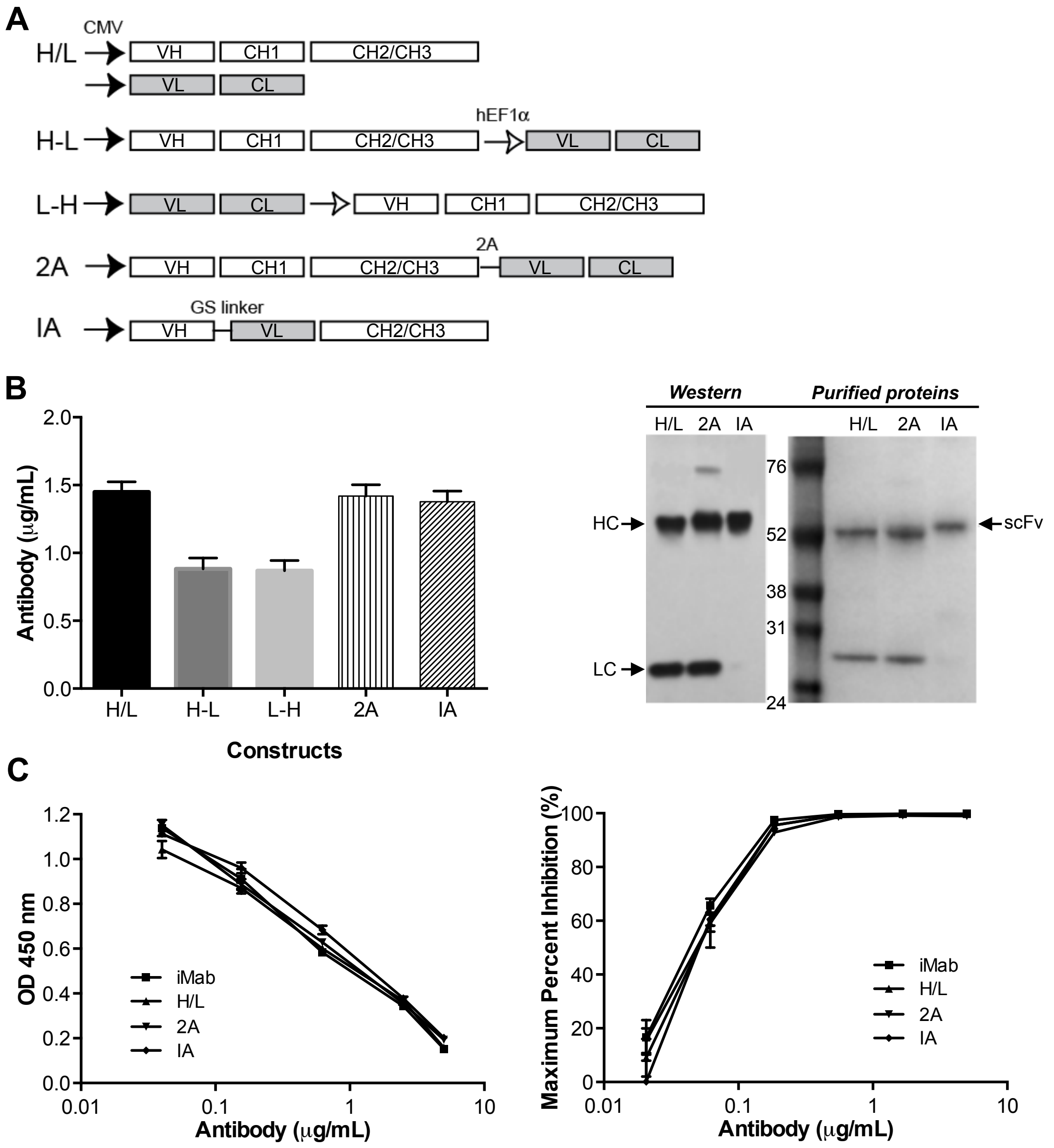

Figure S1

5A8 expression cassettes and *in vitro* characterizations.

(A) Schematic representation of mAb transgene configurations (heavy chain genes shown in white and light chain shown in gray) in the pVAX1 vector. Cytomeglovirus (CMV) and human elongation factor-1 alpha (hEF1α) promoters are indicated by closed black or open white arrows, respectively. The 2A cassette contains H-and L-chain genes separated by a furin cleavage site coupled with a P2A self-processing peptide (2A). The immunoadhesin (IA) contains a single-chain variable fragment (scFv) fused to the Fc region. **(B)** Left panel: *In vitro* 5A8 expression in supernatant 72 hours after pDNA transfection of 293A cells measured by CD4 binding ELISA (mean ± SEM). Right panel: Western blot of cell culture supernatants detected by anti-mouse HRP and mAbs purified from supernatants were resolved by SDS-PAGE and visualized with Coomassie Blue. The faint ~80kDa band detected in the 2A samples by Western blot is unprocessed antibody. Molecular weight ladder represents kDa. **(C)** Left panel: Purified mAbs compete with iMab-HRP for sCD4 binding. Right panel: Purified mAbs neutralize HIV-1 pseudotyped virus tested in TZM-bl cells.

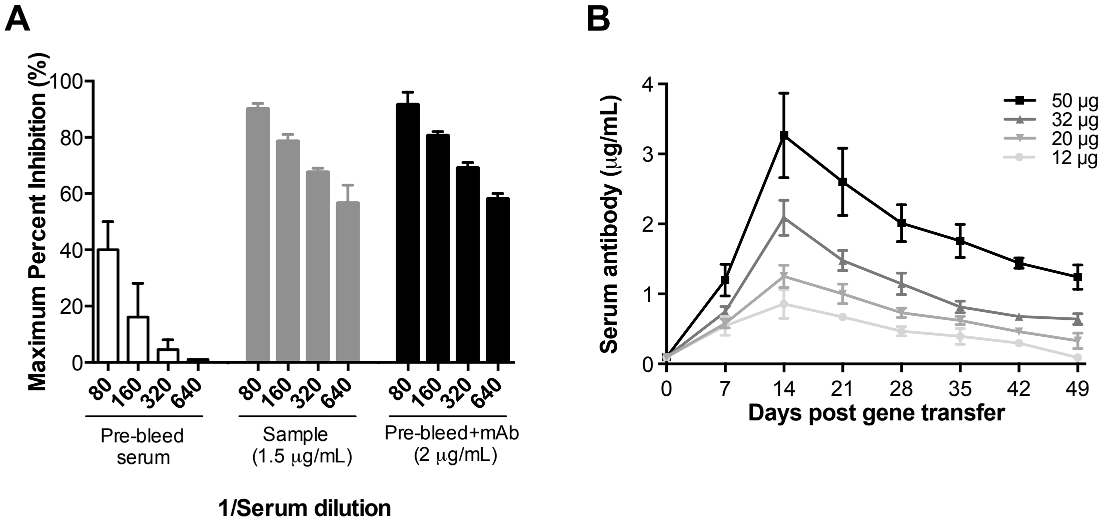

Figure S2

EP delivery of pDNA results in functional dose-dependent serum mAb in mice.

(A) Mice were administered 50 µg H/L 5A8 pDNA in the pVAX1 vector followed by EP. To verify functional activity of the expressed mAb, maximum percent inhibition (MPI) measured by psuedotyped HIV-1 neutralization in TZM-bl cells was compared between serially diluted pre-bleed serum, serum samples taken after DNA/EP, and pre-bleed serum mixed with *in vitro* produced mAb. Mouse serum prior to gene transfer (pre-bleed) was evaluated for antibody neutralization of HIV-1, showing non-specific activity at low serum dilutions. Endogenously expressed mAb retains functional activity comparable to *in vitro* produced mAb. **(B)** mAb expression is pDNA dose-dependent. BALB/c mice (n=6) were injected with 12, 20, 32 or 50 µg of H/L 5A8 pDNA in the pVAX1 vector followed by EP. Serum was collected weekly and mAb concentration was determined by sCD4 binding ELISA. All data are presented as mean ± SEM.

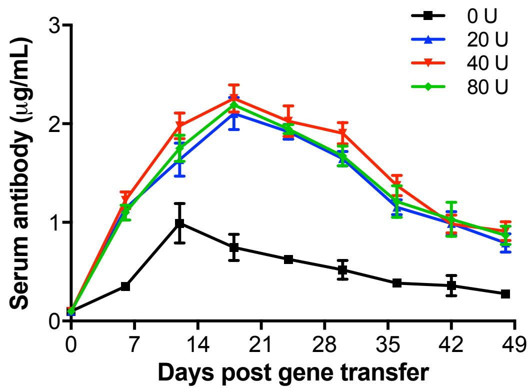

Figure S3

Hyaluronidase pretreatment enhances mAb expression.

BALB/c mice (n=4-5) were pretreated with 20, 40 or 80 U of hyaluronidase or saline (0 U) as a control 2 hours prior to administration of 20 μg of H/L 5A8 pDNA in the pVAX1 vector followed by EP. Serum was collected weekly and mAb concentration was determined by sCD4 binding ELISA. Antibody expression was evaluated by area-under-the curve measurements during the 48-day period studied. All data are presented as mean ± SEM.

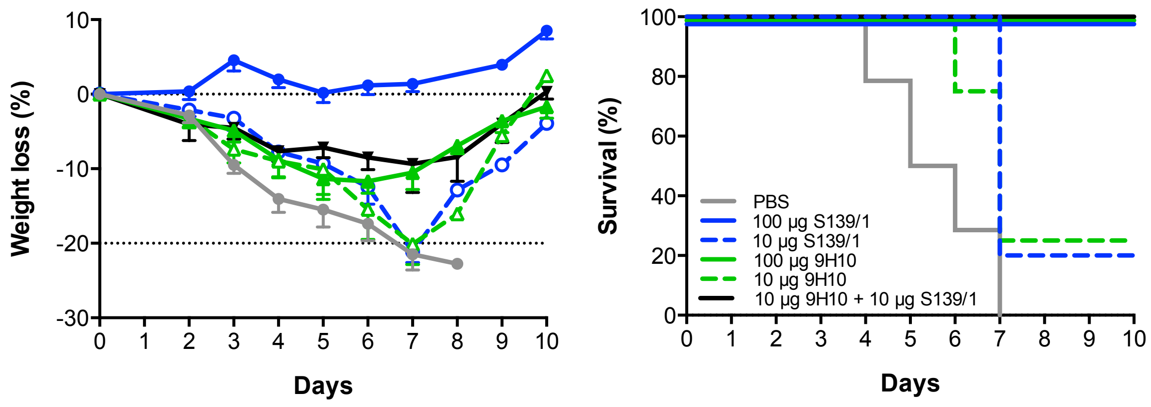

Figure S4

Passive administration of anti-influenza mAbs in mice defines the partially protective dose against influenza challenge.

BALB/c mice (n=3-5) were passively administered mAb via the intraperitoneal route one day prior to influenza challenge. Mice were challenged on day 0 with 13 MLD_{50} of H3N2 (A/Aichi/2/68) delivered intranasally. Left panel: mean weight loss (%) ± SEM compared to day 0. Right panel: survival (%) as depicted in Kaplan-Meier plots following challenge.

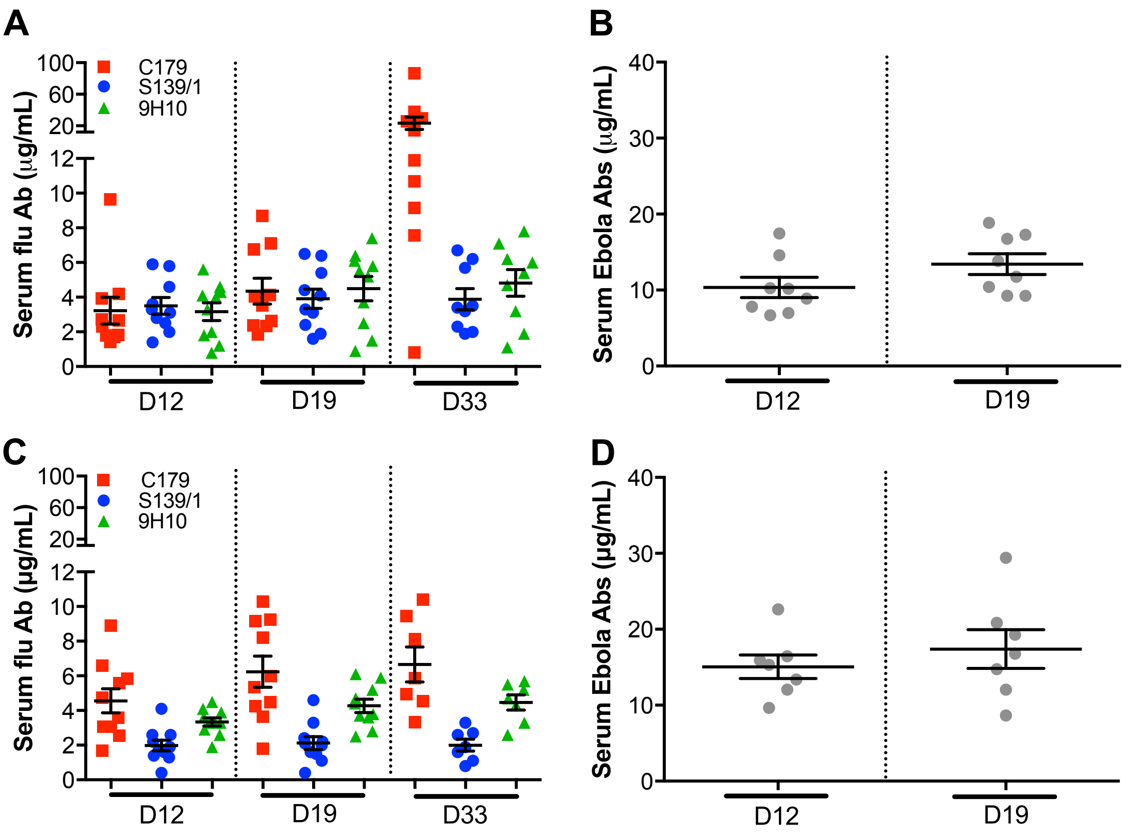

Figure S5

Serum mAb concentrations from mice challenged with group 1 or group 2 influenza viruses.

Mice were administered pDNA followed by EP and challenged as described in Fig. 3. mAb expression from mice challenged with **(A-B)** H1N1 and **(C-D)** H3N2. **(A, C)** Individual mAb concentrations for C179, S139/1 and 9H10. **(B, D)** Total anti-Ebola mAb concentrations. Each symbol represents an individual animal and error bars indicate mean ± SEM.

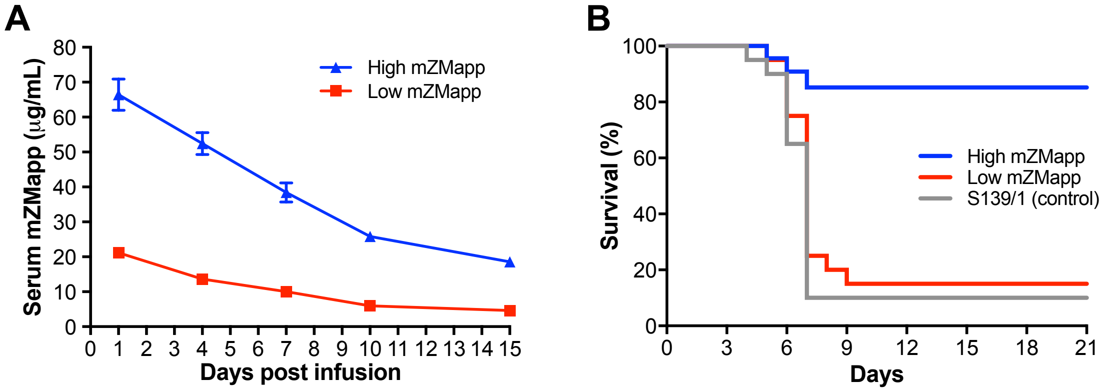

Figure S6

Passive infusion of high-dose mZMapp protects mice from Ebola challenge.

(A) The pharmacokinetic properties of mZMapp were initially evaluated in BALB/c mice. Mice (n=6/group) were infused via the intraperitoneal route with high dose mZMapp (40 µg/mAb; 120 µg total; ~6 mg/kg total) or low dose mZMapp (10 µg/mAb, 30 µg total; ~1.5 mg/kg total) on day 0. Blood was collected at the indicated time points and total mZMapp concentrations were evaluated by GP binding ELISA. Mean ± SEM are shown. **(B)** In a separate experiment, mice (n=20/group) were infused with a low or high dose of mZMapp or an anti-influenza mAb, S139/1 (30 µg total) as a control, one day prior to intraperitoneal challenge with 100 PFU mouse-adapted Ebola virus. Mice were monitored for lethality for 21 days. The Kaplan-Meier plot depicting survival is shown.

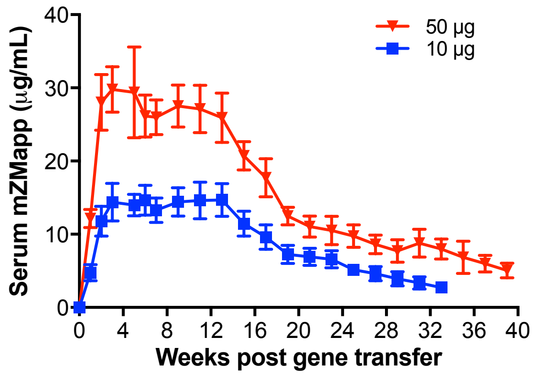

Figure S7

Duration of mZMapp expression following pDNA/EP administration.

BALB/c mice (n=6-9) were pretreated with 20 U of hyaluronidase and administered 10 or 50 μg H/L gWiz pDNA encoding each 13C6, 2G4 and 4G7 at distinct sites followed by EP. Total serum mAb concentrations were measured by GP-binding ELISAs. All data are represented as mean ± SEM.

Supplemental Methods

5A8 / 5A8-like Ab *in vitro* expression

5A8 constructs were evaluated for mAb expression by transient transfection in 293A cells using 5 µg/mL polyethylenimine and 1 µg pDNA. Transfected cells were maintained in Hybridoma-SFM medium for 72 hours. CD4 binding ELISA was used to quantify 5A8 in culture supernatants. Culture supernatants were collected, filtered, purified using Protein G Plus Agarose (Pierce). Eluted mAbs were concentrated by centrifugation (Millipore). mAbs were analyzed by SDS-PAGE visualized with Simply Blue and Western blot detected with anti-mouse HRP.

5A8 assessment for soluble CD4 binding in competition ELISA

In vitro produced 5A8 was assessed for its ability to compete with HRP-labeled iMab for soluble CD4 binding as previously described[1].

HIV-1 pseudovirus-based *in vitro* neutralization assays

Molecular cloned Env-pseudotyped virus G16 was prepared[2] and neutralization was assessed using TZM-bl cells (NIH AIDS Research and Reference Reagent Program, Division of AIDS, NIAID, NIH) with a luciferase-based assay as previously described[1].

Supplemental References

1. Seaman, MS, Janes, H, Hawkins, N, Grandpre, LE, Devoy, C, Giri, A, *et al.* (2010). Tiered categorization of a diverse panel of HIV-1 Env pseudoviruses for assessment of neutralizing antibodies. *Journal of virology* **84**: 1439-1452.
2. Sun, M, Pace, CS, Yao, X, Yu, F, Padte, NN, Huang, Y, *et al.* (2014). Rational design and characterization of the novel, broad and potent bispecific HIV-1 neutralizing antibody iMabm36. *J Acquir Immune Defic Syndr* **66**: 473-483.
